# Feasibility of evaluation of the natural history of kidney disease in the general population using electronic healthcare records

**DOI:** 10.1093/ckj/sfaa175

**Published:** 2020-10-22

**Authors:** Faye Cleary, David Prieto-Merino, Sally Hull, Ben Caplin, Dorothea Nitsch

**Affiliations:** 1 Department of Non-Communicable Disease Epidemiology, Faculty of Epidemiology and Population Heath, London School of Hygiene and Tropical Medicine, London, UK; 2 Clinical Effectiveness Group, Centre for Primary Care and Public Health, Queen Mary University of London, London, UK; 3 Department of Renal Medicine, University College London Medical School, London, UK

**Keywords:** CKD, CKD progression, creatinine, electronic healthcare records, primary care

## Abstract

**Background:**

Knowledge about the nature of long-term changes in kidney function in the general population is sparse. We aim to identify whether primary care electronic healthcare records capture sufficient information to study the natural history of kidney disease.

**Methods:**

The National Chronic Kidney Disease Audit database covers ∼14% of the population of England and Wales. Availability of repeat serum creatinine tests was evaluated by risk factors for chronic kidney disease (CKD) and individual changes over time in estimated glomerular filtration rate (eGFR) were estimated using linear regression. Sensitivity of estimation to method of evaluation of eGFR compared laboratory-reported eGFR and recalculated eGFR (using laboratory-reported creatinine), to uncover any impact of historical creatinine calibration issues on slope estimation.

**Results:**

Twenty-five per cent of all adults, 92% of diabetics and 96% of those with confirmed CKD had at least three creatinine tests, spanning a median of 5.7 years, 6.2 years and 6.1 years, respectively. Median changes in laboratory-reported eGFR (mL/min/1.73 m^2^/year) were −1.32 (CKD) and −0.60 (diabetes). Median changes in recalculated eGFR were −0.98 (CKD) and −0.11 (diabetes), underestimating decline. Magnitude of underestimation (and between-patient variation in magnitude) decreased with deteriorating eGFR. For CKD Stages 3, 4 and 5 (at latest eGFR), median slopes were −1.27, −2.49 and -3.87 for laboratory-reported eGFR and −0.89, −2.26 and −3.75 for recalculated eGFR.

**Conclusions:**

Evaluation of long-term changes in renal function will be possible in those at greatest risk if methods are identified to overcome creatinine calibration problems. Bias will be reduced by focussing on patients with confirmed CKD.

## INTRODUCTION

Chronic kidney disease (CKD) is an irreversible reduction in kidney function that may progress over prolonged time without symptoms. In rare cases, the disease can progress to end-stage renal disease (ESRD) requiring renal replacement therapy (RRT) [1–3]. More common complications preceding ESRD include increased cardiovascular risk, acute kidney injury (AKI), hospital admission and mortality, with increasing risks associated with lower levels of kidney function [4]. Slowing of progression of kidney disease is therefore of great importance to reduce morbidity and burden on healthcare services. Due to its asymptomatic nature, the characteristics of kidney disease progression in the general population from onset to the requirement of dialysis are not well-understood. Improvements in knowledge may lead to better decision-making with potential to delay progression and improve patient outcomes.

In the UK, >99% of the population are registered with a general practitioner (GP), with GPs acting as the gatekeeper to non-emergency specialized care. The National Institute for Health and Care Excellence (NICE) offers evidence-based guidance on managing patients with CKD in primary care and advice on criteria for referral to secondary care. Referral is recommended in a minority of patients including those with CKD Stages 4–5, proteinuria, rapidly declining glomerular filtration rate (GFR), uncontrolled hypertension and genetic renal diseases [3]. In 2004, the Quality and Outcomes Framework was introduced to incentivize long-term condition management in primary care [5]. Performance measures introduced included creatinine testing in patients at high risk of CKD and maintenance of a register of all adults with CKD Stages 3–5 [6, 7]. Recognition of CKD and testing for renal function in primary care has since increased [8].

This article presents the results of a feasibility study investigating whether it is possible to study the natural history of kidney disease using data from primary care electronic healthcare records (EHRs). Using a large database of EHR data extracted in England and Wales in 2015–16, it explores availability of repeat creatinine tests and attempts to describe changes in renal function, within risk factor subgroups. Issues surrounding reliability of estimation of changes in renal function are evaluated, including testing frequency, changes in creatinine calibration practices and gaps in primary care monitoring.

## MATERIALS AND METHODS

### Database

The National Chronic Kidney Disease Audit database was used for analysis. The audit was a cross-sectional study set up to investigate CKD identification and management in primary care in England and Wales in 2014–16 [9, 10]. It evaluated performance of renal function testing in patients at risk of CKD and coding of CKD for patients with established biochemical CKD Stages 3–5, identified by two estimated GFR (eGFR) measures <60 mL/min/1.73m^2^ a minimum of 90 days apart. Data were extracted from 1044 GP practices, for all adult patients alive and registered at the GP practice at data extraction with coded NICE-defined CKD risk factors or at least one creatinine test result recorded between 2008 and data extraction. Data collected included basic demographic characteristics, CKD risk factor codes and all serum creatinine and reported eGFR results recorded between 2008 and data extraction. Age–sex stratified practice list size data and practice ethnicity breakdown were also collected.

### Variables

At the time of the audit, the majority of laboratories reported eGFR using the Modification of Diet in Renal Disease (MDRD) study equation, although this is unlikely to be adjusted for ethnicity which is not typically available to the laboratory. We recalculated eGFR using the MDRD study equation, adjusted for age, sex and ethnicity. CKD Stages 3–5 were identified by two recalculated eGFR measures <60 mL/min/1.73m^2^ a minimum of 90 days apart, with at least one measure recorded in the last 2 years prior to data extraction. CKD stage was identified using recalculated eGFR for the most recently recorded creatinine test. Throughout this article, the term CKD will refer to patients with biochemically confirmed CKD Stages 3–5 unless otherwise stated. Coded CKD Stages 3–5, as defined by a Read code, were also explored in some analyses. Urinary albumin:creatinine ratio (ACR) may also be used to evaluate severity of CKD, although uptake of repeat ACR testing in primary care is low [9], and change in eGFR is more commonly used to identify progression of renal disease.

Risk factors explored were diabetes, hypertension, cardiovascular disease (CVD) and CKD stage. Co-morbidities were defined by the presence of relevant Read codes recorded at any time prior to data extraction. Analyses of hypertension excluded patients with a diabetes code to reduce likelihood of effects being driven by co-occurring diabetes.

Frequency of repeat creatinine tests was defined as the number of creatinine test results recorded for each patient between 2008 and data extraction. Duration of follow-up was difficult to ascertain due to lack of data on time of registration at a GP practice. Duration of coverage of tests was defined as the time between the first and last creatinine test. Loss to follow-up was arbitrarily defined as having no creatinine test in the last 3 years prior to data extraction but with at least three creatinine tests recorded prior. Read codes were used to identify initiation of RRT, and all creatinine test results captured post-initiation of RRT were excluded from analysis.

### Statistical analysis

#### Availability of repeat creatinine tests

The percentage of adults with at least three creatinine tests was summarized by risk factor to evaluate data completeness for estimation of slopes of change in eGFR. Denominators for underlying health condition risk factor groups were determined by summing the number of patients with coded risk factors in the database. Denominators for the entire adult population and age, sex and ethnicity groups were determined using practice list size data. Missing list size for 56 Welsh practices was imputed using the average list size in Wales. In patients with at least three creatinine tests, the frequency of tests, duration of coverage of tests, average time between tests, percentage lost to follow-up and percentage initiating RRT in those lost to follow-up were summarized by risk factor. Potential reasons for gaps in primary care monitoring might be low priority for testing due to good health or management in secondary care due to advancement of disease. Summaries were repeated in patients with diabetes only, with additional stratification by age and sex.

#### Slope estimation

##### Laboratory reporting practices: creatinine calibration and eGFR reporting rules

When serum creatinine blood tests are ordered in UK primary care, laboratories are required to report eGFR if laboratory-specific criteria are met (usually eGFR < 60 or eGFR < 90) corresponding to thresholds of accuracy for GFR-estimating equations. Prior to estimating GFR, creatinine concentrations must be calibrated to an international reference standard (isotope dilution mass spectrometry). In recent years, laboratories have reported correctly calibrated creatinine results, although historically un-calibrated results may have been reported [12, 13]. Creatinine results in the EHR may, therefore, not be comparable within or between patients over time and it may not be straightforward to identify which results were calibrated from the EHR. Coded data extracted from the EHR does not include information on laboratory creatinine calibration practices or eGFR reporting rules and both may vary by laboratory and over time.

**FIGURE 1:**
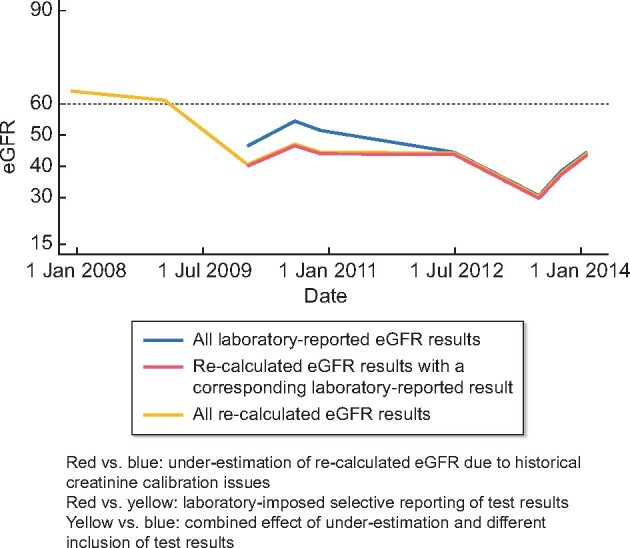
Example profile of laboratory-reported and recalculated eGFR results available for an individual patient in the EHR.

Figure 1 shows an example profile of both laboratory-reported and recalculated eGFR measures over time in an individual patient. In this example, while recently reported and recalculated results are a close match, older recalculated results appear to be underestimated. This is likely due to failure to calibrate historical creatinine results to international standards. Slope analysis using recalculated eGFR may lead to overestimation of slopes (underestimation of rate of decline) if recent measures are more accurate but older measures are underestimated. Also, some recalculated eGFR results do not have a corresponding reported eGFR result due to laboratory reporting conventions and, therefore, slope estimation using reported eGFR may be biased by selective inclusion of test results. Descriptive checks were performed to assess the frequency of reported and recalculated eGFR test results agreeing by +/−1 and by +/−3 mL/min/1.73 m^2^, for all available eGFR results in the database, stratified by calendar year and CKD stage.

Slope of change in eGFR was estimated using linear regression for all patients with at least three valid test results, with separate regression models for each patient. This approach is similar to that used by GPs in routine care to estimate individual changes in kidney function and may be subject to measurement error. Mixed modelling was not used in this analysis, due to concerns about the model assumptions imposed. Analysis was carried out separately for laboratory-reported eGFR and recalculated eGFR to evaluate sensitivity of estimation of slopes to method of evaluation of eGFR and analysis of recalculated eGFR was repeated using only those test results with a corresponding laboratory-reported eGFR to evaluate sensitivity of estimation to laboratory-imposed selective inclusion of test results (Table 1). Values of eGFR outside of the valid range (15–150 mL/min/1.73 m^2^) and an excess of reported eGFR values of 60 and 90 (likely coded in GP records as >60 or >90 but appearing inaccurately in the database simply as 60 and 90) were excluded from analysis.

**Table 1.** eGFR slope regression analysis criteria

Analysis	Test results included
Reported GFR	All laboratory reported eGFR results
MDRD (1)	Recalculated eGFR results for all creatinine test results with a corresponding reported GFR result
MDRD (2)	Recalculated eGFR results for all creatinine test results

#### Comparisons of slope of eGFR

Boxplots of slope of eGFR were stratified by slope estimation method and by risk factor, CKD stage and testing frequency. Only patients with at least three reported eGFR results were included to restrict comparison to the same population. To reduce impact of outliers, whiskers represent 5% and 95% percentiles. Distribution of slopes in all patients with at least three valid recalculated eGFR test results was tabulated for reference, constituting a different population of likely healthier patients.

##### Individual differences in slope estimates

Difference between slope estimates was computed for each patient. Discrepancy between reported and recalculated eGFR slopes using corresponding test results only [Reported GFR—MDRD (1)] shows the effect on slope estimation of creatinine calibration issues. Discrepancy between reported eGFR and recalculated eGFR slopes using all creatinine test results [Reported GFR – MDRD (2)] shows the effect on slope estimation of creatinine calibration issues and laboratory reporting restrictions combined.

Boxplots of the distribution of individual differences in slopes were produced by risk factor and CKD stage. Repeat sensitivity boxplots were stratified by ethnicity (coded black or not) to rule out differences being driven by failures to correct for ethnicity in laboratory-reported results. Descriptive paired *t*-tests were used to identify any statistically significant mean difference in slopes for each comparison by risk factor.

## RESULTS

### Study population

The audit database covered a population of ∼6.5 million adults and was representative of the general population in terms of age and sex. Of the underlying adult population, ∼6% of patients had a diabetes code, 18% had a hypertension code, 6% had a CVD code, 4% had a CKD code and 4% had confirmed CKD.

### Availability of repeat tests

About 2.2 million patients (34%) had at least one creatinine test, 1.6 million (25%) had at least three creatinine tests and 1.1 million (17%) had at least three valid laboratory-reported eGFR results. Approximately 5000 patients (<0.1%) had a code for RRT initiation at any time, with around half of those codes dated prior to 2008 when creatinine data collection began. Of those with a RRT code post-2008, 1583 (60.5%) had at least three GFR tests prior to Read-coded RRT initiation.

Table 2 presents the availability of repeat creatinine tests in all adults and by risk factor. In patients with at least three creatinine tests, the median number of tests was 7, spanning a median of 5.7 years. About 2.4% of these patients had no test performed in the last 3 years of follow-up. Availability of repeat tests and testing frequency was considerably higher and loss to follow-up was lower in high-risk groups, particularly diabetes. Patients lost to follow-up commonly had a RRT code, particularly those with diabetes, coded CKD and eGFR indicating late-stage CKD. For repeat results in diabetes patients, see Supplementary data.

**Table 2.** Availability of repeat creatinine tests in primary care in all adults and by risk factor

Risk factor	Number of patients	Patients with ≥3 tests (*N*, %)	Test frequency^a^ (median + IQR)	Duration of test coverage, years^a^ (median + IQR)	Time (months) between tests^a^ (median + IQR)	No test in last 3 years^a^ (*N*, %)	Coded RRT if no test in last 3 years (*N*, %)
All adults	6 513 000	1 597 629 (24.5%)	7 (5, 10)	5.7 (4.2, 6.4)	8.4 (6.1, 11.2)	39 091 (2.4%)	2.0%
Age							
18–39	2 301 700^b^	59 187 (2.6%)	4 (3, 6)	4.0 (2.5, 5.5)	9.3 (6.2, 13.3)	3 338 (5.6%)	2.6%
40–59	2 214 100^b^	419 144 (18.9%)	5 (4, 8)	5.1 (3.4, 6.1)	9.2 (6.6, 12.5)	14 732 (3.5%)	1.7%
60–79	1 578 600^b^	824 468 (52.2%)	7 (5, 10)	5.9 (4.5, 6.5)	8.4 (6.2, 11.0)	15 960 (1.9%)	2.2%
80+	418 600^b^	294 830 (70.4%)	9 (6, 13)	6.1 (5.0, 6.6)	7.4 (5.4, 9.8)	5 061 (1.7%)	1.9%
Sex							
Male	3 200 400^b^	765 907 (23.9%)	7 (5, 10)	5.7 (4.2, 6.4)	8.4 (6.1, 11.0)	17 634 (2.3%)	2.8%
Female	3 312 600^b^	831 715 (25.1%)	7 (4, 10)	5.7 (4.2, 6.4)	8.5 (6.1, 11.4)	21 457 (2.6%)	1.4%
Ethnicity							
Black	111 300^b^	17 917 (16.1%)	6 (4, 9)	5.2 (3.4, 6.3)	8.5 (6.1, 11.6)	492 (2.7%)	3.7%
Non-black	6 401 700^b^	1 579 712 (24.7%)	7 (5, 10)	5.7 (4.2, 6.4)	8.4 (6.1, 11.2)	38 599 (2.4%)	2.0%
Diabetes	394 568	364 565 (92.4%)	10 (7, 14)	6.2 (5.1, 6.7)	6.6 (5.0, 8.5)	2 053 (0.6%)	14.3%
Hypertension	1 102 781	959 922 (87.0%)	8 (5, 11)	5.9 (4.7, 6.5)	8.2 (6.0, 10.6)	16 000 (1.7%)	3.9%
CVD	390 506	351 273 (90.0%)	9 (6, 13)	6.1 (5.0, 6.6)	7.4 (5.4, 9.6)	3 362 (1.0%)	7.6%
CKD code	266 358	251 792 (94.5%)	11 (7, 15)	6.2 (5.2, 6.7)	6.3 (4.6, 8.5)	3 495 (1.4%)	20.0%
Confirmed CKD	256 568	247 352 (96.4%)	10 (7, 15)	6.2 (5.2, 6.7)	6.4 (4.6, 8.7)	N/A^c^	N/A^c^
CKD stage^d^ (last GFR)							
1 (90+)	456 902	319 127 (69.8%)	6 (4, 9)	5.5 (3.9, 6.4)	8.7 (6.4, 11.5)	7 657 (2.4%)	0.03%
2 (60–90)	1 342 474	937 219 (69.8%)	7 (4, 9)	5.6 (4.1, 6.4)	8.9 (6.6, 11.7)	24 636 (2.6%)	0.1%
3 (30–60)	371 893	318 931 (85.8%)	9 (6, 13)	6.0 (4.7, 6.6)	7.0 (5.0, 9.4)	5 831 (1.8%)	1.0%
4 (15–30)	19 016	18 137 (95.4%)	15 (9, 21)	6.3 (5.3, 6.8)	4.6 (3.2, 6.4)	334 (1.8%)	51.2%
5 (<15)	4 293	3 743 (87.2%)	13 (8, 21)	5.5 (3.3, 6.6)	4.0 (2.7, 6.1)	605 (16.2%)	88.6%

a In patients with ≥3 tests.

b Population age, sex and ethnicity breakdown are estimated based on aggregate data provided at the practice level.

c Loss to follow-up not evaluable in confirmed CKD since group definition requires creatinine measurement in last 2 years.

d CKD stage evaluated in all patients with at least one creatinine test result (34% of all adults).

### Comparison of slopes of eGFR

Descriptive checks showed better agreement between reported and recalculated eGFR for more recent measures and for later stages of CKD. Slope of recalculated eGFR was estimated in 1.6 million patients and slope of reported eGFR was estimated in 1.1 million patients. The duration of coverage of tests for slope analyses and percentage agreement statistics are provided in the Supplementary data.

**FIGURE 2:**
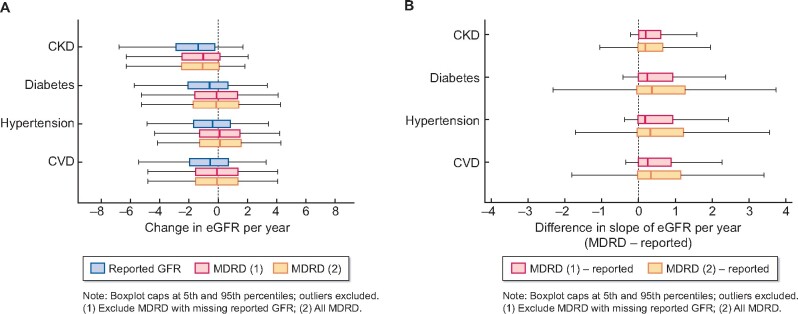
Distribution of slopes of change in eGFR (A) and distribution of differences between recalculated and reported GFR slopes (B) in patients with at least three reported eGFR results, by risk factor and method of estimation of slope of eGFR.

Figure 2A shows the distribution of slopes of change in eGFR by risk factor and slope estimation method, among all patients with at least three reported GFR results. The median slope varies by risk factor and estimation method and is consistently higher for analyses using recalculated eGFR than for analysis of reported eGFR. The median slope of recalculated eGFR using all creatinine tests [MDRD (2)] was consistently higher in the population of patients with at least three recalculated eGFR results (a more complete population, not shown) than in those with at least three reported GFR results. (For numerical figures and for population breakdown by age, sex and ethnicity, see Supplementary data.)

Figure 2B shows the distribution of individual differences in slope estimates. Descriptive paired *t*-tests showed strong statistical significance for a non-zero mean difference in slopes for all slope comparisons by risk factor, P < 0.001. Positive differences show systematic overestimation of slope of change in eGFR (underestimation of decline) when using recalculated eGFR compared with reported GFR results, with a median overestimation of ∼0.2 mL/min/1.73 m^2^/year across subgroups for comparison using the same test results [MDRD (1)—Reported], increasing to ∼0.3 mL/min/1.73 m^2^/year, for comparison not restricted to the same test results [MDRD (2)—Reported]. Discrepancies are lower in CKD than in other risk groups. Ninety-five per cent of differences between ‘MDRD (1)’ and ‘Reported GFR’ slope estimates in CKD patients lie between −0.25 and 1.6 mL/min/1.73 m^2^/year, which may not be clinically important.

About 1.1% of patients with at least three reported eGFR test results had coded black ethnicity. Sensitivity analysis excluding patients with coded black ethnicity (i.e. for which laboratory ethnicity correction is not required) had no effect on observed overestimation. (For boxplots, see Supplementary data.)

**FIGURE 3:**
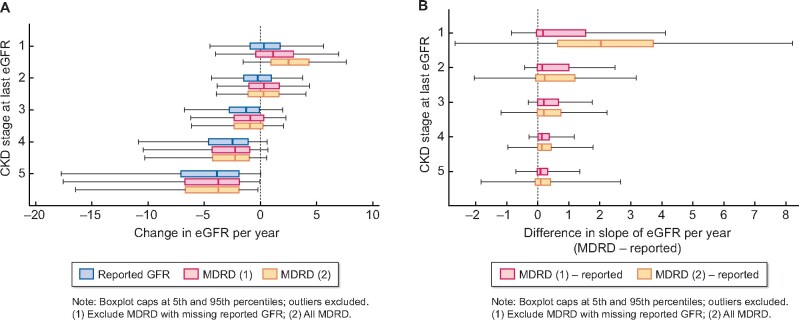
Distribution of slope of change in eGFR (A) and distribution of differences between recalculated and reported GFR slopes (B) in patients with at least three reported eGFR results, by CKD stage (1–5) at most recent measure and method of estimation of slope of eGFR.

Figure 3A shows the distribution of slopes by CKD stage. As expected, slope of decline is steeper in patients reaching later stages of disease. There is greater variation in slopes at later stages of disease, with some patients appearing to decline much more rapidly than others. Figure 3B shows discrepancy in slopes between estimation methods by CKD stage. Discrepancy diminishes considerably as kidney function worsens and slope estimation is highly sensitive to the estimation method for patients with latest eGFR in the normal range.

Comparison of slopes by frequency of tests (see Supplementary data) showed markedly reduced variability for patients with five or more tests, likely due to increased precision of estimation for increasing number of tests, although plausibly driven by patients with worse kidney function having more tests.

## DISCUSSION

The aim of this study was to identify whether it may be feasible to study the natural history of kidney disease using EHRs held in UK primary care. The database used was large and representative of the UK population. While testing frequency was low in the general population, high-risk groups were tested regularly, sufficient to study long-term longitudinal changes in renal function. It is possible that we may not capture a representative sample of CKD patients if the sickest patients are managed solely in secondary care throughout creatinine data collection. Informative loss to follow-up from primary care may also be a concern, although rates of loss to follow-up were low, particularly in diabetes.

A major issue that may compromise evaluation of longitudinal changes in renal function using primary care EHRs is lack of creatinine calibration to international standards in historical results reported by laboratories [11, 12]. It is very challenging to identify when calibration practices may have changed from the EHR but failure to correct historical measures would mean that measures are not comparable within or between patients, and estimates of change in eGFR will be overestimated in many patients (underestimating decline). One solution may be to use laboratory-reported eGFR but lower values are more likely to be reported than those in (or closer to) the normal range, leading to selective inclusion of test results. It may be possible to develop statistical methods capable of identifying the time point(s) in individuals at which creatinine calibration practices have changed and apply appropriate correction factors to un-calibrated results. Some authors have attempted to do this [13]. Another approach would be to restrict analysis to test results reported post-2012, when calibration issues are less common, but this would impact duration of follow-up. Restricting analysis to patients with CKD would also reduce overestimation to levels that may not be of clinical importance.

This study did not consider the possibility of temporary losses in renal function that may occur due to an acute event. Although scheduled annual review tests in primary care are likely to be carried out on a relatively stable population, tests may also be carried out on patients who present to their GP due to ill health and it is not known how many AKI events may be captured in primary care eGFR data. Longer-term drops in renal function following an acute event are also possible and changes over time may be non-linear in some patients [4], which may require a more complex modelling approach. Slope distributions reported in this article may, therefore, not be clinically reliable.

Primary care EHRs in the UK are an excellent source of data on changes in renal function over a long duration of follow-up in patients at the highest risk of CKD and CKD progression. Future studies aiming to study longitudinal changes in renal function should take care to handle data quality issues present in EHRs. In particular, failure to account for creatinine calibration problems may lead to underestimation of decline in renal function over time. The study population should be selected taking into account data availability and reliability of analytical methods. Future studies may also need to account for informative loss to follow-up. We recommend the use of joint modelling of longitudinal changes and the drop-out process [14] with linkage to external databases to help establish reasons for loss to follow-up.

## SUPPLEMENTARY DATA

Supplementary data are available at *ckj* online.

## Supplementary Material

sfaa175_supplementary_data
