# Supplementary material for: Feasibility of evaluation of the natural history of kidney disease in the general population using electronic healthcare records
Source: Clin Kidney J. 2020 Oct 22;14(6):1603–9. doi: 10.1093/ckj/sfaa175 (PMC8162846; doi:10.1093/ckj/sfaa175)
Supplement: sfaa175_supplementary_data [file sfaa175_supplementary_data.docx]

**Supplementary Materials**

Tables and figures referred to in the manuscript are provided in the supplementary materials, including descriptive data checks, sensitivity analyses, analysis population characteristics and numerical figures to support graphics provided in the manuscript.

**Table 1** Availability of repeat creatinine tests in primary care in adults with coded diabetes

| **Risk factor** | **Patients with**  **≥3 tests**  **(N, %)** | **Test frequency***  **(median + IQR)** | **Duration of test coverage, years***  **(median + IQR)** | **No test in last 3 years***  **(N, %)** | **Coded RRT if no test in last 3 years***  **(N,%)** |
| --- | --- | --- | --- | --- | --- |
| All diabetes | 366,098 (92.5%) | 10 (7, 14) | 6.2 (5.1, 6.7) | 2,154 (0.6%) | 13.7% |
| Age:  18-40  40-60  60-80  80+ | 1,3871 (69.8%)  96,139 (89.2%)  193,926 (95.3%)  62,162 (95.9%) | 6 (4, 8)  8 (6, 11)  11 (8, 14)  12 (9, 17) | 4.6 (2.8, 6)  5.8 (4.1, 6.5)  6.3 (5.5, 6.7)  6.4 (5.9, 6.8) | 263 (1.9%)  899 (0.9%)  750 (0.4%)  242 (0.4%) | 5.3%  10.5%  21.1%  12.4% |
| Sex:  Male  Female | 205,549 (92.1%)  160,549 (92.9%) | 10 (7, 14)  10 (7, 14) | 6.2 (5, 6.7)  6.2 (5.2, 6.7) | 1,257 (0.6%)  897 (0.6%) | 15.1%  11.8% |
| Hypertension | 222,608 (95.7%) | 11 (8, 15) | 6.3 (5.6, 6.8) | 1065 (0.5%) | 23.8% |
| CVD | 92,641 (95.9%) | 12 (9, 16) | 6.4 (5.7, 6.8) | 396 (0.4%) | 33.3% |
| CKD code | 69,095 (97.2%) | 14 (10, 19) | 6.5 (6, 6.9) | 437 (0.6%) | 62.7% |
| Confirmed CKD | 67,105 (98.5%) | 14 (10, 19) | 6.5 (5.9, 6.9) | N/A** | N/A** |
| CKD stage***  (last GFR):  1 (90+)  2 (60-90)  3 (30-60)  4 (15-30)  5 (<15) | 100,569 (89.8%)  181,391 (94.1%)  75,590 (97.1%)  7,032 (97.7%)  1,516 (93.2%) | 9 (6, 12)  10 (7, 13)  13 (9, 18)  18 (13, 25)  16 (10, 24) | 5.9 (4.3, 6.6)  6.2 (5.1, 6.7)  6.4 (5.8, 6.8)  6.6 (5.9, 6.9)  6.0 (3.9, 6.7) | 775 (0.8%)  843 (0.5%)  238 (0.3%)  88 (1.3%)  210 (13.9%) | 0%  0.8%  10.1%  77.3%  93.8% |

*in patients with **≥**3 tests

**Loss to follow up not evaluable in confirmed CKD since group definition requires creatinine measure in last 2 years

***CKD stage evaluated in all diabetes patients

**Table 2** Duration of coverage of tests for patients with at least 3 test results (and hence meeting slope analysis criteria)

| **Duration of coverage of tests** | **≥ 3 valid reported**  **eGFR results**  **[Reported GFR; MDRD (1)]**  **N (%)** | **≥3 valid MDRD re-calculated**  **eGFR results**  **[MDRD (2)]**  **N (%)** | **≥ 3 creatinine results**  **N (%)** |
| --- | --- | --- | --- |
|  | N = 1,100,460 | N = 1,594,629 | N = 1,597,629 |
| <90 days | 5,136 (0.5%) | 5,033 (0.3%) | 5,038 (0.3%) |
| 90 days – 1 year | 28,117 (2.6%) | 28,883 (1.8%) | 29,014 (1.8%) |
| 1-2 years | 67,739 (6.2%) | 74,653 (4.7%) | 74,791 (4.7%) |
| 2-4 years | 224,007 (20.4%) | 258,104 (16.2%) | 258,470 (16.2%) |
| 4-6 years | 410,626 (37.3%) | 573,623 (36.0%) | 574,211 (35.9%) |
| 6-8.5 years | 364,835 (33.1%) | 654,378 (41.0%) | 656,105 (41.1%) |

*Valid refers to GFR results in the 0-150 range

**Figure 1** Percentage agreement (+/-1 and +/-3 ml/min/1.73m^2^) between reported eGFR and corresponding re-calculated MDRD eGFR by calendar year and eGFR CKD stage

**Table 3** eGFR regression slopes by risk factor and coverage of underlying population

**A** – Re-calculated eGFR slopes (in all patients with at least 3 valid re-calculated eGFR results)

| **Risk factor** | **Number and percent of patients analysed from full extracted dataset** | **Change in eGFR per year**  **(median + IQR)**  **[MDRD-2]** |
| --- | --- | --- |
| Diabetes | 363,626 (92.2%) | +0.10 (-1.61, 1.84) |
| Hypertension | 737,472 (84.7%) | +0.30 (-1.23, 1.94) |
| CVD | 350,938 (89.9%) | +0.07 (-1.49, 1.68) |
| CKD code | 251,784 (94.5%) | -0.47 (-1.90, 0.85) |
| Confirmed CKD | 247,351 (96.4%) | -1.04 (-2.48, 0.11) |

**B** – All slopes (in all patients with at least 3 REPORTED eGFR results)

| **Risk factor** | **Number and percent of patients analysed from full extracted dataset** | **Change in eGFR per year**  **(median + IQR)**  **[REPORTED GFR]** | **Change in eGFR per year**  **(median + IQR)**  **[MDRD-1]** | **Change in eGFR per year**  **(median + IQR)**  **[MDRD-2]** |
| --- | --- | --- | --- | --- |
| Diabetes | 253,974 (64.4%) | -0.60 (-2.07, 0.68) | -0.11 (-1.62, 1.33) | -0.13 (-1.72, 1.41) |
| Hypertension | 523,638 (60.1%) | -0.39 (-1.70, 0.84) | +0.09 (-1.28, 1.50) | +0.12 (-1.29, 1.58) |
| CVD | 272,624 (69.8%) | -0.56 (-1.97, 0.70) | -0.09 (-1.54, 1.33) | -0.09 (-1.58, 1.38) |
| CKD code | 244,127 (91.7%) | -0.79 (-2.23, 0.45) | -0.45 (-1.87, 0.86) | -0.49, (-1.91, 0.81) |
| Confirmed CKD | 241,047 (94.0%) | -1.32 (-2.84, -0.16) | -0.98 (-2.44, 0.18) | -1.04, (-2.48, 0.11) |

**C** – Difference in slopes (in all patients with at least 3 REPORTED GFR results)

| **Risk factor** | **Number and percent of patients analysed from full extracted dataset** | **Difference in slopes**  **(median + IQR)**  **[MDRD (1) - REPORTED GFR]** | **Difference in slopes**  **(median + IQR)**  **[MDRD (2) - REPORTED GFR]** |
| --- | --- | --- | --- |
| Diabetes | 253,974 (64.4%) | +0.24 (-0.01, 0.94) | +0.36 (-0.06, 1.27) |
| Hypertension | 523,638 (60.1%) | +0.18 (-0.02, 0.94) | +0.32 (-0.04, 1.22) |
| CVD | 272,624 (69.8%) | +0.25 (-0.01, 0.90) | +0.34 (-0.04, 1.15) |
| Confirmed CKD | 241,047 (94.0%) | +0.19 (0.00, 0.61) | +0.17 (-0.03, 0.65) |

**Table 4** Age, sex, and ethnicity breakdown by underlying health condition in patients with at least 3 REPORTED GFR results (reflects population of boxplots in manuscript Figure 2), contextualised by age, sex, ethnicity breakdown of the underlying population, for patients with at least 3 creatinine tests, and among all patients with at least 3 reported GFR results

| **Risk factor** | **Underlying population under study*** | **All adults**  **≥3 creatinine** | **All adults**  **≥3 reported GFR** | **CKD** | **Diabetes** | **Hypertension** | **CVD** |
| --- | --- | --- | --- | --- | --- | --- | --- |
| Number of adults | N = 6,513,000 | N = 1,597,629 | N = 1,100,460 | N = 241,047 | N = 253,974 | N = 523,638 | N = 272,624 |
| Median + IQR last re-calculated eGFR | N/A | 73 (62, 86) | 68 (58, 78) | 48 (40, 54) | 69 (55, 81) | 69 (57, 79) | 65 (53, 77) |
| Age  18-39  40-59  60-79  80+ | 2,301,700 (35.3%)  2,214,100 (34.0%)  1,578,600 (24.2%)  418,600 (6.4%) | 59,187 (3.7%)  419,144 (26.2%)  824,468 (51.6%)  294,830 (18.4%) | 16,340 (1.5%)  224,735 (20.4%)  605,442 (55.0%)  253,943 (23.1%) | 945 (0.4%)  14,036 (5.8%)  115,208 (47.8%)  110,858 (46.0%) | 2,733 (1.1%)  47,329 (18.6%)  148,065 (58.3%)  55,847 (22.0%) | 3,012 (0.6%)  87,394 (16.7%)  292,425 (55.8%)  140,807 (26.9%) | 314 (0.1%)  22,318 (8.2%)  150,435 (55.2%)  99,557 (36.5%) |
| Sex:  Male  Female | 3,200,400 (49.1%)  3,312,600 (50.9%) | 765,907 (47.9%)  831,715 (52.1%) | 503,054 (45.7%)  597,402 (54.3%) | 98,402 (40.8%)  142,645 (59.2%) | 134,891 (53.1%)  119,083 (46.9%) | 227,455 (43.4%)  296,182 (56.6%) | 156,648 (57.5%)  115,975 (42.5%) |
| Ethnicity:  Black  Non-black | 111,300 (1.7%)  6,401,700 (98.3%) | 17,917 (1.1%)  1,579,712 (98.9%) | 12,638 (1.2%)  1,087,822 (98.8%) | 1,026 (0.4%)  240,021 (99.6%) | 4,457 (1.8%)  249,517 (93.2%) | 5,312 (1.0%)  518,326 (99.0%) | 1,466 (0.5%)  271,158 (99.5%) |

IMPORTANT: Percentages are of column headers (in this case, population under study or underlying health condition)
*Population age, sex and ethnicity breakdown is estimated based on aggregate data provided at the practice level

In patients with confirmed CKD that have at least 3 reported eGFR results, only 0.4% are aged under 40 and 93.8% are aged 60 and over. There is under-representation of males and black ethnicity in this group compared to the underlying population. In diabetes, 1.1% are aged under 40 and 80.3% are aged 60 and over, covering a broader population than for confirmed CKD. Black ethnicity appears to be under-represented across all risk factor subgroups, when compared to the underlying population and considering that co-morbidity prevalence is likely truly higher in black ethnicity than non-black ethnicity. The same may be true for males, who suffer higher burden of co-morbidities than females.

**Table 5** Age, sex, ethnicity breakdown by CKD stage (1-5) at last GFR in patients with at least 3 REPORTED GFR results (reflects population of boxplots in manuscript Figure 3), contextualised by age, sex, ethnicity breakdown of the underlying population, for patients with at least 3 creatinine tests, and among all patients with at least 3 reported GFR results

| **Risk factor** | **Underlying population under study*** | **All adults**  **≥3 creatinine** | **All adults**  **≥3 reported GFR** | **Last GFR**  **CKD stage 1** | **Last GFR**  **CKD stage 2** | **Last GFR**  **CKD stage 3** | **Last GFR**  **CKD stage 4** | **Last GFR**  **CKD stage 5** |
| --- | --- | --- | --- | --- | --- | --- | --- | --- |
|  | N = 6,513,000 | N = 1,597,629 | N = 1,100,460 | N = 75,435 | N = 701,579 | N = 302,199 | N = 17,751 | N = 3,471 |
| Last re-calculated eGFR result (median + IQR) | N/A | 73 (62, 86) | 68 (58, 78) | 96 (92, 102) | 72 (67, 79) | 52 (45, 56) | 25 (22, 28) | 11 (8, 13) |
| Age  18-39  40-59  60-79  80+ | 2,301,700 (35.3%)  2,214,100 (34.0%)  1,578,600 (24.2%)  418,600 (6.4%) | 59,187 (3.7%)  419,144 (26.2%)  824,468 (51.6%)  294,830 (18.4%) | 16,340 (1.5%)  224,735 (20.4%)  605,442 (55.0%)  253,943 (23.1%) | 1,788 (2.4%)  24,320 (32.2%)  40,526 (53.7%)  8,801 (11.7%) | 12,799 (1.8%)  175,110 (25.0%)  404,968 (57.7%)  108,702 (15.5%) | 1,403 (0.5%)  23,444 (7.8%)  151,978 (50.3%)  125,374 (41.4%) | 199 (1.1%)  1,158 (6.5%)  6,328 (35.7%)  10,066 (56.7%) | 144 (4.2%)  696 (20.1%)  1,638 (47.2%)  993 (28.6%) |
| Sex:  Male  Female | 3,200,400 (49.1%)  3,312,600 (50.9%) | 765,907 (47.9%)  831,715 (52.1%) | 503,054 (45.7%)  597,402 (54.3%) | 41,861 (55.5%)  33,574 (44.5%) | 329,663 (47.0%)  371,916 (53.0%) | 121,692 (40.3%)  180,507 (59.7%) | 7,815 (44.0%)  9,936 (56.0%) | 2,014 (58.0%)  1,457 (42.0%) |
| Ethnicity:  Black  Non-black | 111,300 (1.7%)  6,401,700 (98.3%) | 17,917 (1.1%)  1,579,712 (98.9%) | 12,638 (1.2%)  1,087,822 (98.8%) | 3,701 (4.9%)  71,734 (95.1%) | 7,443 (1.1%)  694,136 (98.9%) | 1,297 (0.4%)  300,902 (99.6%) | 109 (0.6%)  17,642 (99.4%) | 84 (2.4%)  3,387 (97.6%) |

IMPORTANT: Percentages are of column headers (in this case, population under study or CKD stage indicated by last re-calculated GFR result)
*Population age, sex and ethnicity breakdown is estimated based on aggregate data provided at the practice level

There appears to be under-representation of males, black ethnicity and young adults for last GFR results that indicate CKD stage 3. While under-representation of young adults may be partially due to low prevalence of kidney disease, this is unlikely the case for males and black ethnicity. Under-representation of these groups appears to diminish as CKD progresses. Some of these groups appear to be over-represented at CKD stage 5, possibly suggesting worse outcomes for groups that are not identified at earlier stages of disease.

**Figure 2** Distribution of slopes of change in eGFR (A) and distribution of differences between re-calculated and reported GFR slopes (B) in patients with at least 3 reported GFR results, by risk factors and method of estimation of slope of eGFR, separate plots for non-black (1) and black (2) ethnicity

**1A 1B**

**
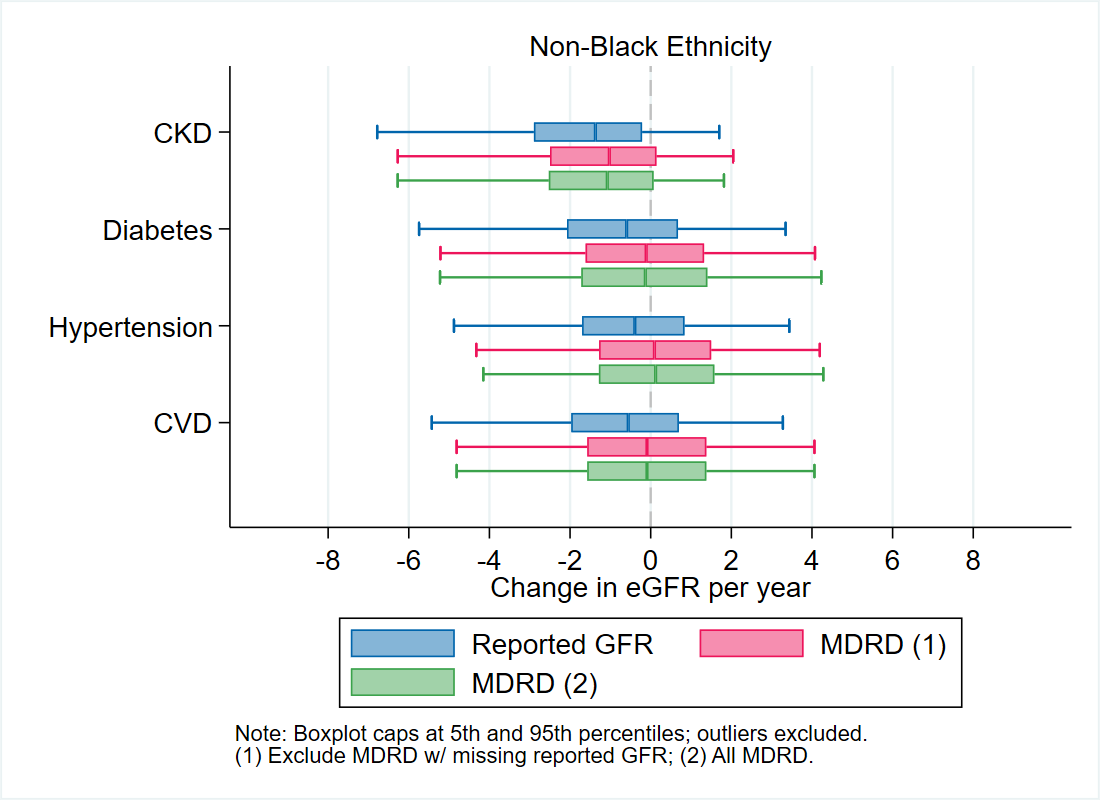

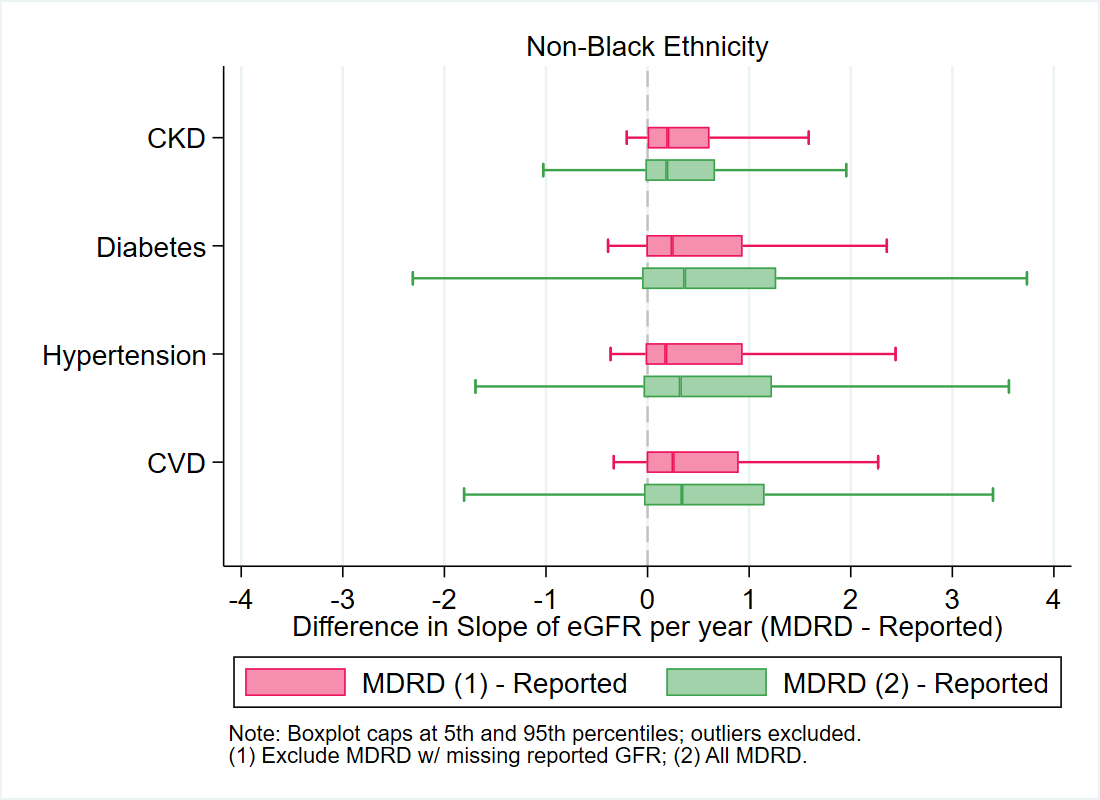
**

**2A 2B**

**
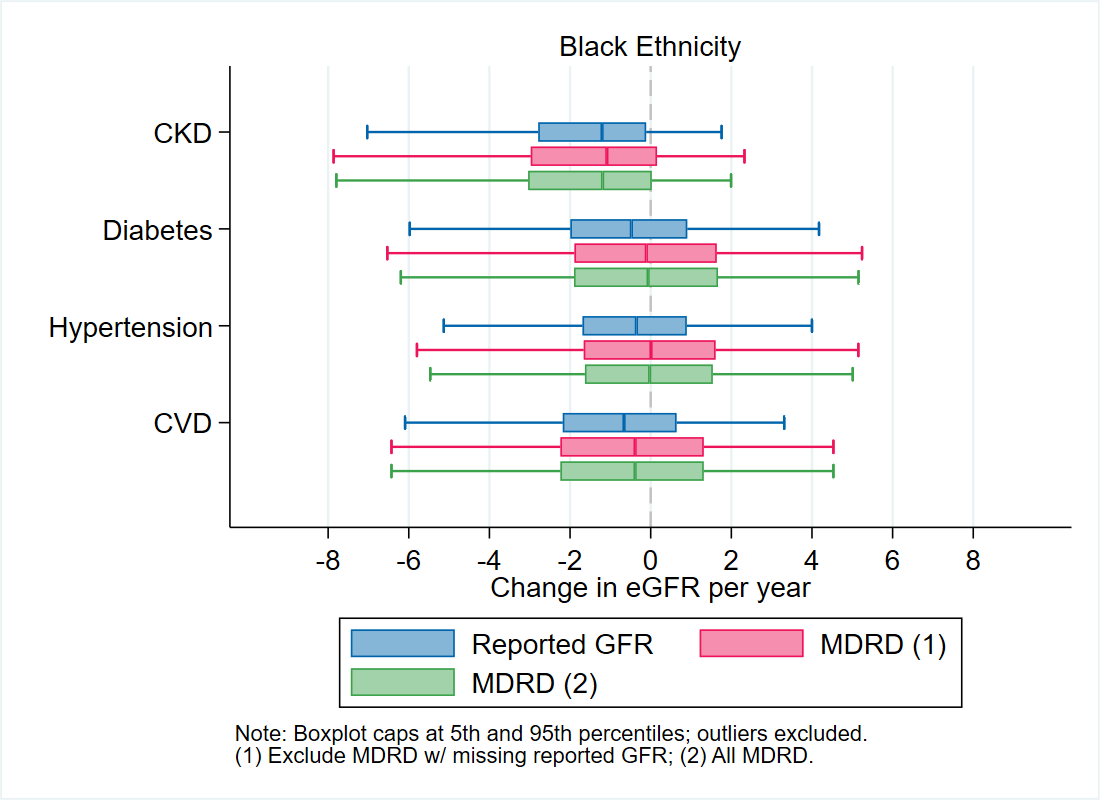

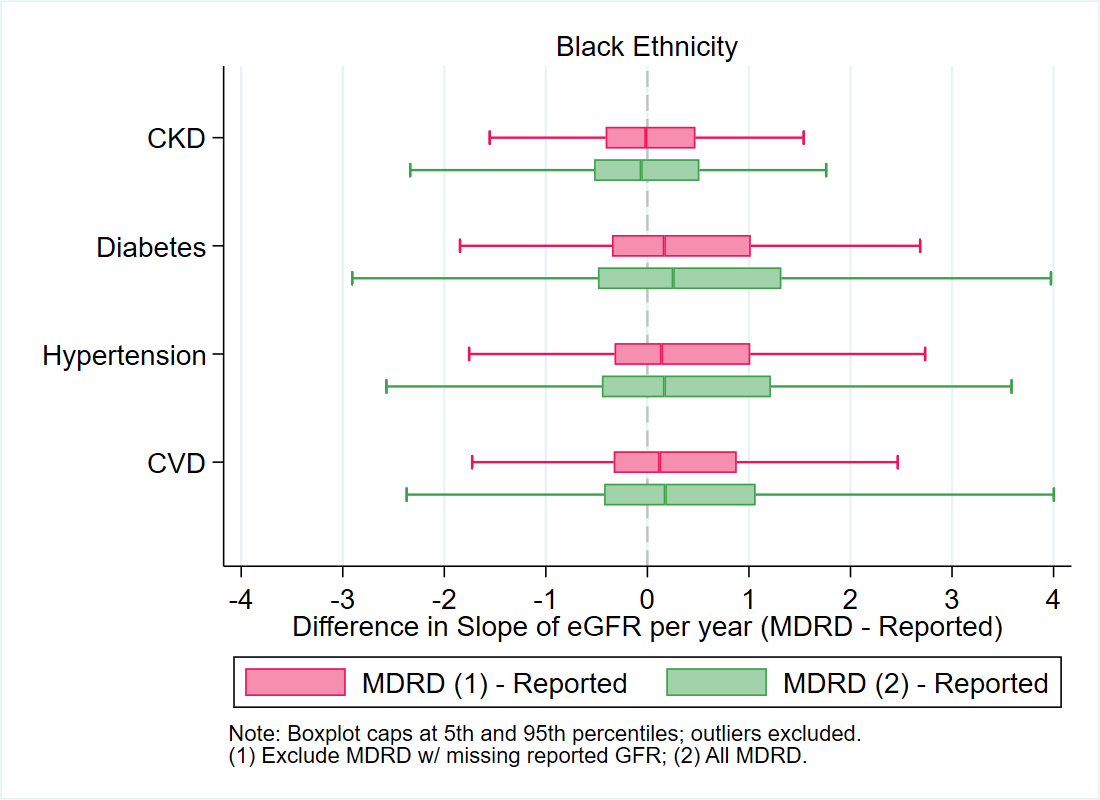
**

**Figure 3** Distribution of slope of change in eGFR by test frequency

**
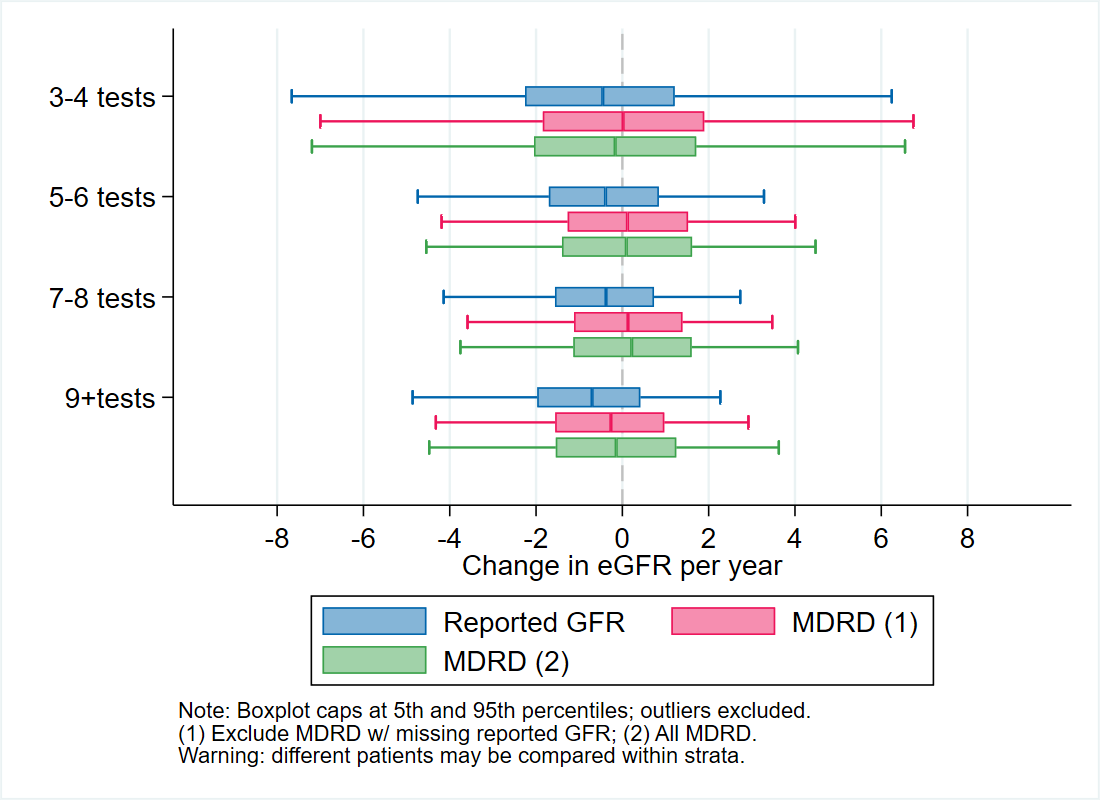
**
